# Combined Effect of a Locking Plate and Teriparatide for Incomplete Atypical Femoral Fracture: Two Case Reports of Curved Femurs

**DOI:** 10.1155/2015/213614

**Published:** 2015-05-26

**Authors:** Hiroyuki Tsuchie, Naohisa Miyakoshi, Tomio Nishi, Hidekazu Abe, Toyohito Segawa, Yoichi Shimada

**Affiliations:** ^1^Ugo Municipal Hospital, 44-5 Otomichi, Nishomonai, Ugo 012-1131, Japan; ^2^Department of Orthopedic Surgery, Akita University Graduate School of Medicine, 1-1-1 Hondo, Akita 010-8543, Japan

## Abstract

In surgical treatment for atypical femoral fractures (AFFs), reconstruction nail fixation is recommended for both complete and incomplete fractures. Although it has been reported that AFF is affected by many factors, The ASBMR Task Force 2013 Revised Case Definition of AFFs states that a curved femur is often seen in Asian patients. It is sometimes difficult to insert a nail into a femur in incomplete AFF patients with severely curved femurs. We report two incomplete bisphosphonate-related AFF patients with marked femoral curvatures treated by locking plates and teriparatide, showing early bone unions and favorable long-term outcomes.

## 1. Introduction

Although bisphosphonates (BPs) are the gold standard for osteoporosis pharmacotherapy, several adverse effects related to their long-term use have recently been reported, such as osteonecrosis of the jaw (ONJ) and atypical low-energy subtrochanteric and diaphyseal femoral fractures due to severely suppressed bone turnover (SSBT) [1, 2]. These fractures are typically diagnosed as atypical femoral fractures (AFFs). In surgical treatment, reconstruction nail fixation is recommended for both complete and incomplete fractures [3]. However, in incomplete AFF patients with severely curved femurs, it is difficult to insert a nail into the femur.

We describe herein two patients with incomplete AFFs treated by locking plates and teriparatide, showing favorable courses after surgery.

## 2. Case Presentation

**Case 1 .**

**Figure 1:**
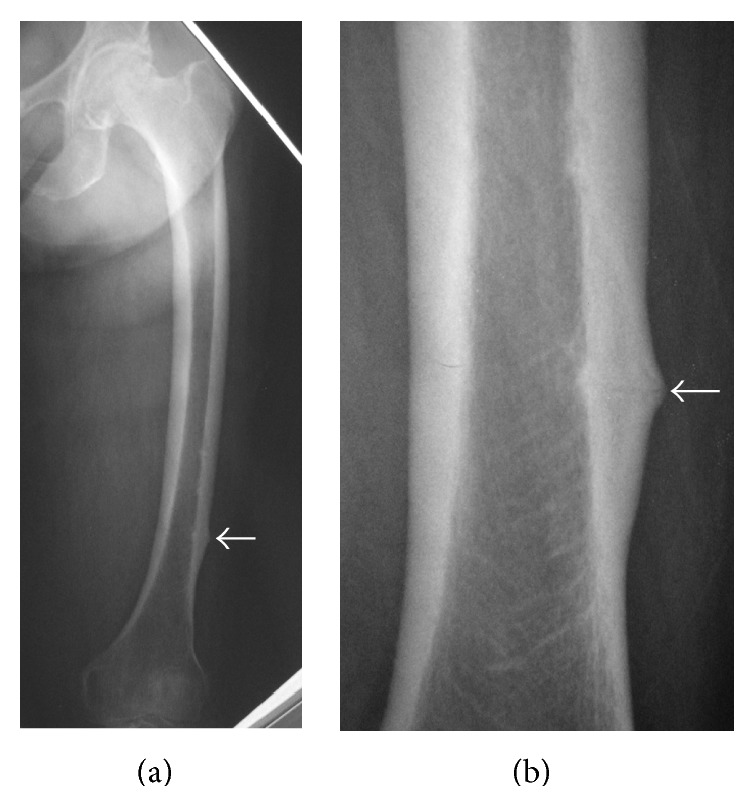
Anteroposterior radiographs of the left femur. (b) is a magnified view of (a). They showed some thickening of the femoral cortex and a horizontal line resembling a fracture over the outer cortical bone of the distal diaphysis.

A 78-year-old woman presented to our outpatient clinic with pain of the left thigh persisting for 1 month. She had no history of trauma. The pain was insidious at onset and gradually increased, and she could not walk for a week by herself. She had been treated for osteoporosis and taken alendronate for 4 years. Plain radiographs showed some thickening of the femoral cortex and a horizontal line resembling a fracture over the outer cortical bone of the left femoral distal diaphysis (Figure 1). Using the method to measure femoral curvature established by Sasaki et al. [4], curvature of the lateral femur was marked: lateral curvature was 12 degrees, and anterior curvature was 11 degrees. On magnetic resonance imaging (MRI), laterally thickened areas of the left femur showed a low signal intensity on T1-weighted images and high signal intensity on T2-weighted images. The bone mineral density (BMD) of the lumbar spine (L2-4, 0.681 g/cm^2^, *T*-score: −2.8 S.D.) and proximal femur (0.641 g/cm^2^, *T*-score: −3.2 S.D.), as assessed by dual-energy X-ray absorptiometry, confirmed the presence of osteoporosis. On laboratory examinations, serum calcium (Ca: 9.1 mg/dL, normal range, 7.8–10.1 mg/dL), inorganic phosphorus (IP: 3.5 mg/dL, normal range, 2.5–4.5 mg/dL), cross-linked N-telopeptide of type I collagen in serum (NTX: 12.8 nmolBCE/L, normal range, 10.7–24.0 nmolBCE/L), bone-specific alkaline phosphatase (BAP: 10.9 *μ*g/L, normal range, 3.8–22.6 *μ*g/L), and serum 25(OH)D (22 ng/mL, normal range, 7–41 ng/mL) were all within their normal ranges. We suspected incomplete AFF of the left femur caused by long-term bisphosphonate therapy and a laterally curved femur, so we instructed her to stop taking alendronate.

**Figure 2:**
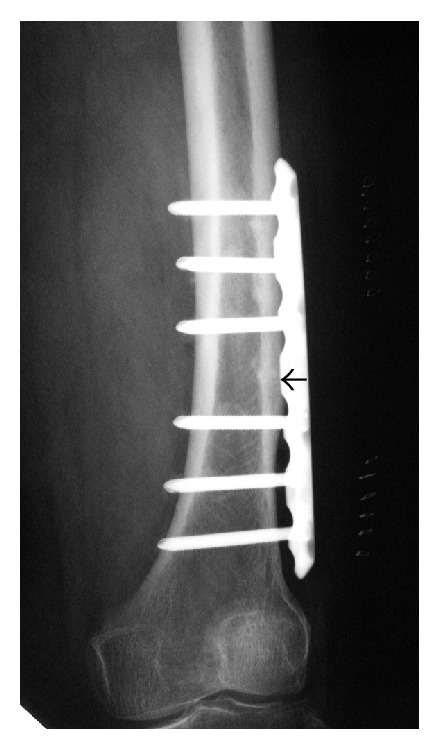
Anteroposterior radiograph of the left femur treated surgically using a locking plate. Black arrow shows original horizontal line.

**Figure 3:**
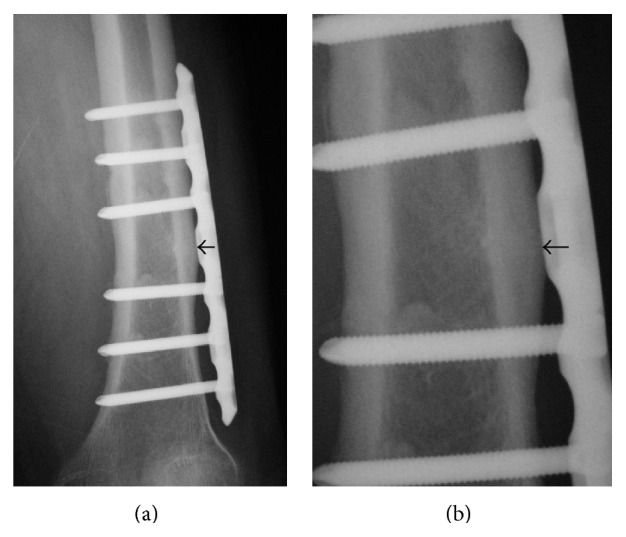
Anteroposterior radiographs of the left femur 3 months after surgical treatment. (b) is a magnified view of (a). The horizontal line over the outer cortical bone (black arrows) had almost disappeared.

The patient was treated surgically using locking plates to prevent complete fracture of the femur (Figure 2). We did not make a skin incision above the fracture region, and we placed a locking plate by sliding it along the femur. After the operation, we started treatment with teriparatide. The postoperative course was uneventful, and she was able to walk without pain 2 weeks after the operation. The horizontal line over the outer cortical bone had almost disappeared 3 months after surgery (Figure 3). Teriparatide administration was finished 1 year after the operation, and SERM (selective estrogen receptor modulator) intake was started after teriparatide treatment. She did not have any pain of the left thigh at the most recent follow-up of 3 years postoperatively.

**Case 2 .**

**Figure 4:**
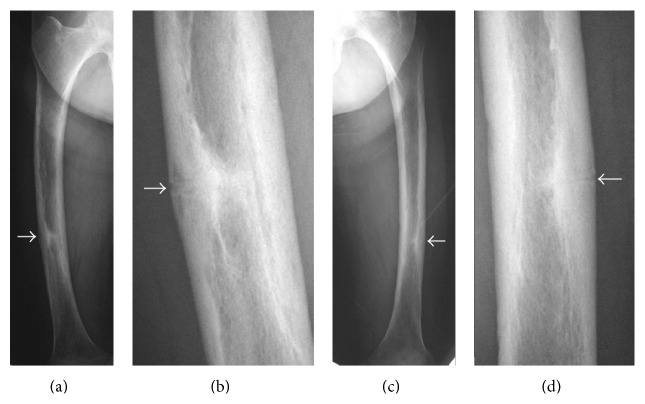
Anteroposterior radiographs of the bilateral femurs ((a) right femur; (c) left femur). (b) and (d) are magnified views of (a) and (c), respectively. They showed thickening of the outer cortex and a horizontal line resembling a fracture.

**Figure 5:**
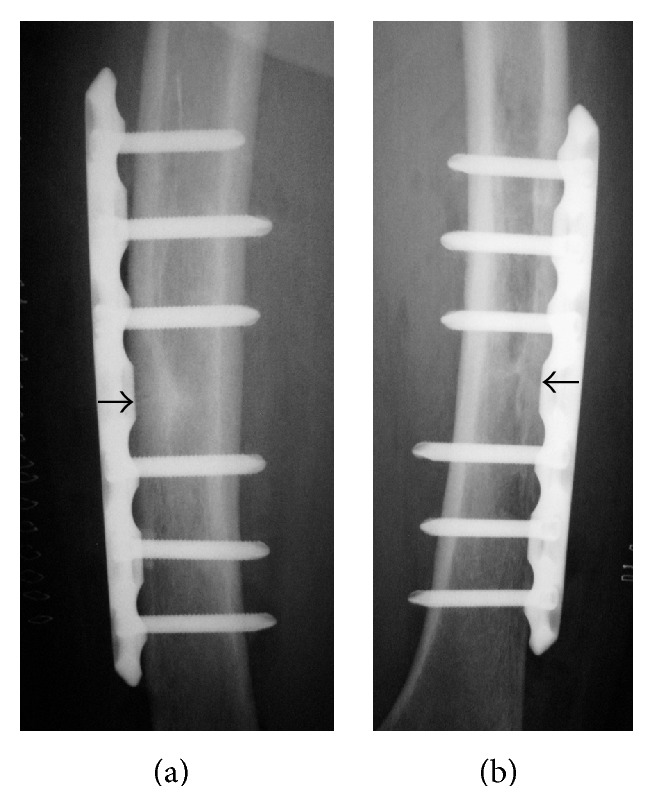
Anteroposterior radiographs of the bilateral femurs treated surgically using locking plates. Black arrows show original horizontal line.

**Figure 6:**
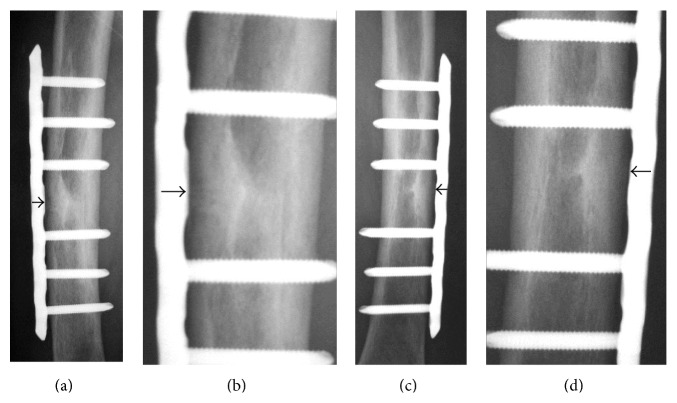
Anteroposterior radiographs of the bilateral femurs ((a) right femur; (c) left femur) 6 months after surgical treatment. (b) and (d) are magnified views of (a) and (c), respectively. The horizontal lines over the outer cortical bone (black arrows) had almost disappeared.

A 77-year-old woman presented to our outpatient clinic with pain of the bilateral thighs persisting for 6 months. The pain was insidious at onset and gradually increased, and she had been unable to walk by herself for 2 weeks. She had been treated for osteoporosis and taken alendronate for 6 years. Plain radiographs of the bilateral femora showed thickening of the outer cortex and a horizontal line resembling a fracture (Figure 4). Curvature of the lateral femur was marked: lateral curvature for right femur 17 degrees and left femur 12 degrees and anterior curvature for right femur 15 degrees and left femur 15 degrees. BMD of the lumbar spine (L2-4, 0.724 g/cm^2^, *T*-score: −2.4 S.D.) and proximal femur (0.559 g/cm^2^, *T*-score: −2.1 S.D.) did not show the presence of osteoporosis. Laboratory examination data (Ca: 9.3 mg/dL, IP: 3.5 mg/dL, NTX: 12.6 nmolBCE/L, and BAP: 14.5 *μ*g/L) were all within their normal ranges. The patient was treated surgically using locking plates, and we instructed her to stop taking alendronate and start teriparatide treatment (Figure 5). She was able to walk without pain 3 weeks after surgery. The horizontal line over the outer cortical bone had almost disappeared 6 months after surgery (Figure 6). Teriparatide administration was finished 1 year after the operation, and eldecalcitol intake was started after teriparatide treatment. She did not have any pain of the bilateral thighs at the most recent follow-up of 2 years postoperatively.

## 3. Discussion

We consider that the present patients had AFFs because they were consistent with the definition although we did not conduct bone histomorphometrical analysis [3]. Although the prognosis varies markedly depending on the presence of complete or incomplete fracture in bisphosphonate-related AFFs, reconstruction nail fixation is recommended for AFF patients on surgical treatment for both complete and incomplete fractures [3]. Although the bone union period after intramedullary nail fixation surgery for normal femoral fracture is approximately 3 months [5], Egol et al. stated that approximately 8.3 months were needed for bone union for bisphosphonate-related complete AFFs [6]. In addition, bone union is sometimes not achieved, and multiple surgeries were required for nonunion patients [4, 6, 7]. On the other hand, the course of bone union after nail fixation surgery for incomplete AFF is favorable. Oh et al. stated that the bone union period after nail fixation surgery for incomplete AFF was approximately 14.3 weeks, and it was possible to achieve bone union in all patients [8]. However, it is difficult to insert a nail into a femur in incomplete AFF patients with severely curved femurs, and complete fracture is sometimes caused on placing an intramedullary nail [8]. Bone union may be delayed if this occurs. We used locking plates for the two incomplete AFF patients because of marked femoral curvatures. Since early load is possible, as in the present cases, we can expect to perform rehabilitation after surgery equivalent to nail fixation. Although nail fixation leads to intramedullary damage, a locking plate does not cause such damage. So, a locking plate is more favorable, and it may be more effective for bone union compared to nail fixation. It was possible to achieve early bone union and favorable long-term outcomes in the present patients.

Although it has been reported that AFF is affected by many factors, The ASBMR Task Force stated that a curved femur is often seen in Asian patients [3]. Sasaki et al. investigated curvatures of the lateral and anterior femurs in AFF cases and found that curvature in AFF patients was significantly greater than that in a control group [4]. The present patients also had bisphosphonate-related AFFs accompanied by a curved femur. Although we previously considered that lumbar kyphosis and deficiency of vitamin D were one of the factors causing femoral curvature, the detailed cause of femoral curvature has not been clarified [9].

In conclusion, we present two incomplete AFF patients showing a favorable course after being treated by locking plate surgery and prescribed teriparatide. Although reconstruction nail fixation may be the first choice of surgical treatment for AFF, locking plate use is one of the options for incomplete AFF patients when we expect the intramedullary nail procedure to be difficult.
